# Using brain-computer interfaces: a scoping review of studies employing social research methods

**DOI:** 10.1186/s12910-019-0354-1

**Published:** 2019-03-07

**Authors:** Johannes Kögel, Jennifer R. Schmid, Ralf J. Jox, Orsolya Friedrich

**Affiliations:** Institute of Ethics, History and Theory of Medicine, LMU Munich, Lessingstr. 2, D-80336 Munich, Germany

**Keywords:** Brain-computer interfaces, Neuroethics, Empirical research, Quantitative methods, Qualitative methods, User experience

## Abstract

**Background:**

The rapid expansion of research on Brain-Computer Interfaces (BCIs) is not only due to the promising solutions offered for persons with physical impairments. There is also a heightened need for understanding BCIs due to the challenges regarding ethics presented by new technology, especially in its impact on the relationship between man and machine. Here we endeavor to present a scoping review of current studies in the field to gain insight into the complexity of BCI use. By examining studies related to BCIs that employ social research methods, we seek to demonstrate the multitude of approaches and concerns from various angles in considering the social and human impact of BCI technology.

**Methods:**

For this scoping review of research on BCIs’ social and ethical implications, we systematically analyzed six databases, encompassing the fields of medicine, psychology, and the social sciences, in order to identify empirical studies on BCIs. The search yielded 73 publications that employ quantitative, qualitative, or mixed methods.

**Results:**

Of the 73 publications, 71 studies address the user perspective. Some studies extend to consideration of other BCI stakeholders such as medical technology experts, caregivers, or health care professionals. The majority of the studies employ quantitative methods. Recurring themes across the studies examined were general user opinion towards BCI, central technical or social issues reported, requests/demands made by users of the technology, the potential/future of BCIs, and ethical aspects of BCIs.

**Conclusions:**

Our findings indicate that while technical aspects of BCIs such as usability or feasibility are being studied extensively, comparatively little in-depth research has been done on the self-image and self-experience of the BCI user. In general there is also a lack of focus or examination of the caregiver’s perspective.

**Electronic supplementary material:**

The online version of this article (10.1186/s12910-019-0354-1) contains supplementary material, which is available to authorized users.

## Background

Research on Brain-Computer Interfaces (BCIs) has seen a rapid development in the last 10 years [1]. A BCI is defined as a device that measures activity of the brain or central nervous system and converts these signals into artificial output [2]. BCIs thus detect and process brain and nervous system activity in order to control and direct external devices, such as personal computers, robotic arms, wheelchairs, or to activate a person’s own muscles [3–8]. A large area of application is in the use of BCIs in combination with medical devices, in order to increase communication or motor control for persons with physical impairments. BCIs have also been implemented in neuro-rehabilitation to improve neurological conditions such as motor paralysis after stroke or spinal cord injury [9, 10], as well as epilepsy [11]. Research has demonstrated that there are many uses for BCIs, including medical uses such as the treatment of psychiatric conditions, as well as non-medical uses such as neuro-enhancement or in gaming products [12–14].

Relevant brain activity from BCI users can be detected either with non-invasive (mostly with electroencephalography (EEG)) or invasive methods [6, 9, 15–17]. Three types of brain activity production and use by the technology can be distinguished [18]. (1) *Passive BCIs* use brain activity not voluntarily produced by the person, such as mental workload or affective states [18]. (2) *Reactive BCIs* are based on changes in brain activity which occur as a result of an individual’s voluntarily focused attention on a specific external stimulus (mostly visual, but also auditory or somatosensory) among a multitude of irrelevant stimuli [5, 18]. (3) *Active BCIs* require that the user applies a certain mental strategy (e.g. imagining moving a limb) [5, 18]. In virtually all BCI types, users receive real-time feedback on their brain activity output, through visual (most common), auditory, tactile, vestibular, or proprioceptive feedback [5]. Certain closed-loop applications of BCIs also apply stimulation to the brain by using electrodes which have been implanted in the brain, for instance in the treatment of epilepsy, or psychiatric disorders [19].

To understand BCI beyond its technical components and medical applications, significant social research is needed to grasp BCI in its practical and human dimension. Insights into the use of BCI and its impact on the user are necessary to develop the relevant knowledge and tools for ethical and legal evaluation.

The manner in which subjectivity or identity is molded by technology is an important issue for many different human-machine interactions, but especially in BCI applications (e.g. [20–22]). Entirely new characteristics involved in BCI, for instance incorporated technologies such as implants, or self-reliant and environment co-creating technologies, have the potential to influence the future of human-machine interaction. BCIs often employ these breaking threshold technologies and therefore present a crucial area of research in the molding of subjectivity through mediation with technology, and in human-machine interactions. Thus, it is clearly an essential matter to understand the first person perspective of BCI users, as well as the outlook of potential users and other BCI stakeholders.

In the interest of gaining an overview of existing studies, we have conducted a scoping review of published studies related to BCI that make use of social research methods. This scoping review is “aimed at mapping key concepts, types of evidence, and gaps in research related to” the field of BCI research “by systematically searching, selecting and synthesizing existing knowledge” [23]. The following paper should serve as a basis for future empirical studies and inform conceptual and ethical deliberations. We include studies considering various BCI stakeholders as well as quantitative and qualitative methods of social research.

## Methods

A comprehensive review of the literature regarding brain-computer-interfaces and social research methods was conducted by following the five stages outlined by Arksey and O’Malley [24]. According to Levac et al. [25], we integrated further aspects such as a tabularly summaries and qualitative thematic analysis.

We aim to identify and characterize key social aspects of BCI use. In particular, the intent of the paper is to retrieve and synthesize existing scientific data on the perspective of BCI users. The focus of the review is not on technological and medical issues, but on psycho-social, personal, and ethical aspects of BCI use. For that purpose we turn to studies that employ social research methods as these methods can be regarded as a standard for reliable research outcomes with respect to societal and psycho-social practices. Hence, we selected the method of a scoping review with the intent of exploring the extent of research on the topic, summarizing findings and identifying research gaps. Given that the body of literature available is quite extensive and heterogeneous, a scoping review of the social and human implications of BCI use helps create a comprehensible overview for future research in this area of emerging and urgent relevance. This means, at the same time, that the body of literature is “not amenable to a more precise systematic review” [26].

### Sources

Six databases were systematically searched and analyzed, namely PubMed, EMBASE, PSYCINFO, PSYNDEX, Sowiport, and SocINDEX due to the wide coverage offered in BCI-oriented research (across fields of medicine, psychology, and social sciences.) From March 1st to May 3rd 2017, two researchers performed the search. Publications dated later than May 3rd 2017 were not included in the systematic research, but may be referred to in the discussion section of the paper. All existing articles which met our research criteria were screened. In light of the novelty of BCI technology we expected further forms of publications and thus identified additional literature through searches in local university libraries, other internet sources, and by cross-referencing. We have drafted an internal review protocol which was not registered.

### Inclusion/Exclusion criteria

Following a research consultation, we agreed on the following rationale for searching the databases. Two primary search terms and seven cross-references were used:

- Search terms:
  - ○brain-computer* OR brain-machine*
- Cross-references with:
  - ○ case study OR empirical OR interview OR qualitative OR quantitative OR questionnaire OR survey.

The cross-references were used to identify research on BCI that utilise social research methods. The search was performed on “all search terms” or “MeSH” terms (Pubmed).

“Methods of social research are the *technical practices* used to identify research questions, collect and analyze data, and present findings” [27]. Social research methods can be differentiated into quantitative and qualitative methods. The latter aim at the interpretation of meanings people assign to their actions, the former seeks to discover regularities in social phenomena. Quantitative methods are associated with surveys, experimental methods and the use of statistics. Qualitative methods often make use of interviews, focus groups, case studies or ethnographies, as well as methods of coding, theme, discourse or narrative analysis [27].

We limited the search in this case to the most relevant terms for the review. General key terms that cover the broad spectrum of research methods (empirical, quantitative, qualitative) were combined with specific terms to prioritize data gathering methods (i.e. case studies, interviews, questionnaires, and surveys).

The search was limited to literature published in English or German, two major scientific languages spoken by researchers in the field. No restriction was applied regarding the date of publication up to the date of the search.

To illustrate this procedure, one electronic search strategy conducted via PSYCINFO we will describe here in detail. The primary search term ‘brain-computer*’ was combined with the cross-reference ‘empirical’ using the AND-function. No specific field was selected. Furthermore, the search options were adjusted by choosing the search mode ‘Find all my search terms’ and selecting the publication type ‘All’. Initiating the search resulted in 25 hits. This procedure was repeated for each search term (brain-computer* and brain-machine*) and each of the seven cross-references separately. In total the research in PSYCINFO yielded 188 hits.

### Analysis

**Fig. 1:**
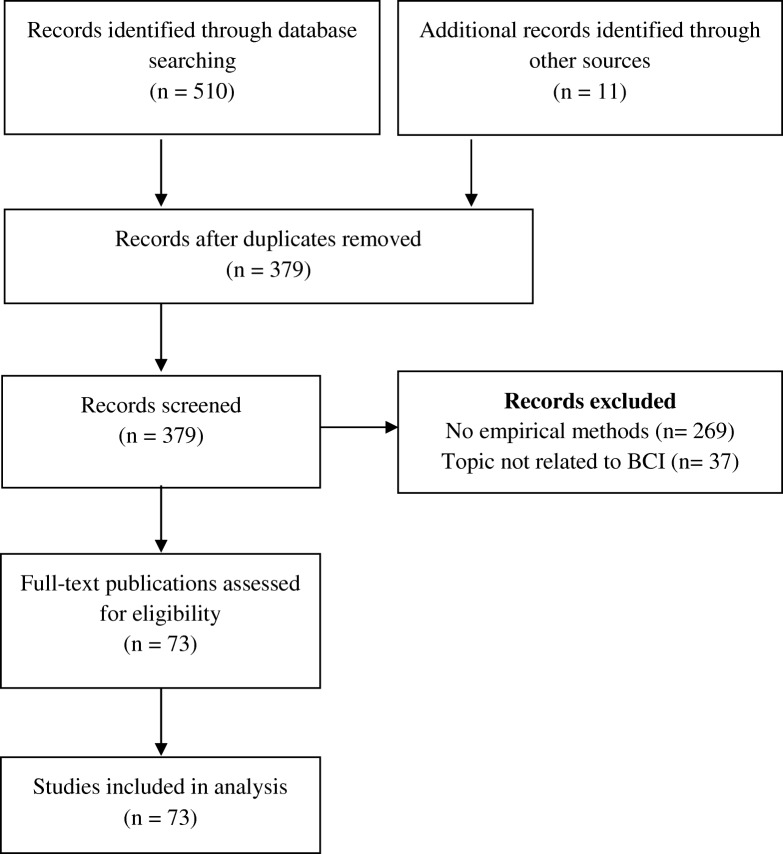
The review process

Figure 1 provides an overview of the review procedure according to the PRISMA flow diagram [28]. The initial database research yielded 510 records. In addition, further publications found online or via a snow-balling method were manually added (*n* = 11) at the first stage of the research process. After removing duplicates and applying the inclusion/exclusion criteria, we identified 73 relevant publications. All of these publications were retrieved as full texts and analyzed using thematic analysis. The results were summarized in an Excel file. Each selected publication has been summarized and listed in tabular form (see Additional file 1).^1^ We assessed risk of bias through evaluating and discussing our findings within a team of researchers at regular intervals.

The studies were categorized according to subgroups, reflecting the different groups of study participants (potential users, actual users, professionals, caregivers). Due to the amount of studies with potential and actual users, these groups each were divided further into two subgroups according to the methods used in the studies (namely quantitative and qualitative methods). This process allowed for a comprehensible display of the results and a more concise comparison within and among the various groups. In a second step, themes were identified by means of inductive reasoning in the style of the Qualitative Research Synthesis approach from Claire H. Major and Maggi Savin-Baden [29]. The themes are displayed either as columns in the tables or as bullet points.

## Results

The final synthesis of the search undertaken contains a total of 73 publications of which 45 report quantitative studies, and ten refer to qualitative studies. Eighteen studies used a mixed-methods approach encompassing both quantitative and qualitative methods. Of the 73 publications, 13 report on the perspectives of potential users, i.e. persons who may have had a particular interest in using BCIs, but had no actual experience with BCIs. Fifty-eight publications focus on persons who have experienced BCIs either before or during the very study of the respective publication. Five studies additionally encompass caregivers/relatives. Seven studies address experts/professionals.

As outlined above, we obtained four groups of user studies (categorized along the axes – quantitative/qualitative studies and potential/actual users): quantitative studies with potential users (*n* = 11), qualitative studies with potential users (*n* = 2), quantitative studies with actual users (*n* = 48), and qualitative studies with actual users (*n* = 21). Studies employing mixed methods fall into both the quantitative and qualitative categories. Because of differences in terms of expectations and the impact of BCI among the users, the last category (qualitative studies with actual users) requires further differentiation. Accordingly, results of non-impaired users and users with physical impairments were assessed separately. The latter often are referred to in the BCI literature as “end-users”. (This terminology, however, could be quite confusing as not all BCI applications e.g. in the case of gaming, aim for persons with physical impairments as their end-users.)

Seventy-one of the 73 publications are focused on (potential or actual) BCI users. Two studies address BCI experts only. However, many of these 71 studies also encompass caregivers, experts, health care professionals or other BCI stakeholders.

### Potential users

Research studies which have been conducted with potential users of BCIs mainly focus on assessing expectations that this particular group has about BCI as a so-called “assistive technology” device. These studies thus mostly limit these expectations to the question of the usability of the technology. The objective of such studies is to obtain information about the profile of the end-users for whom BCIs are being developed. The intent implicit therein is also to draw the developers’ attention to aspects deemed important by potential users, in order to meet user demand and adapt the technology accordingly.

#### Quantitative studies

There are 11 studies included in the review which assess the opinion of potential BCI users by means of quantitative methods (surveys/questionnaires) (Table 1). Three of the included quantitative studies evaluate non-impaired participants. They assess preferences of gamers [12], test for control aspects in gaming [30], and work on selection strategies for BCI use [31]. Eight studies in contrast focused on the needs of persons with physical impairments. The impairment- or medically-focused studies evaluated the interest in BCIs among potential users, marking their preferences regarding different features and functions, and requirements the technology is supposed to meet.

**Table 1:** Studies with potential users employing quantitative research methods

Publication	Interest in BCIs	User preferences regarding BCI functions/features	Expectations towards BCI technology	Other aspects
Ahn et al., 2014 [12]		active and reactive BCIs		high potential of BCI; most potential fields: rehabilitation, prosthesis, gaming
Blabe et al., 2015 [100]		communication	ease of use, high performance, little maintenance, decent aesthetics	
Collinger et al., 2013 [101]		arm/hand and bladder/bowel function	independent use, convenient use, non-invasiveness, functions, costs, set-up time	
Huggins et al., 2011 [88]	high, even for implants		accuracy, speed, simplicity, standby mode	
Huggins et al., 2015 [102]	high among persons with low functional independence	dry electrodes	better speed and set-up time	
Lahr et al., 2015 [103]	high, even for implants		knowledge about risks/rewards	
Kageyama et al., 2014 [89]	depending on severity of disease	communication	various control functions (TV, bed, emergency alarm)	
Pedrocchi et al., 2013 [104]			improve autonomy, home use, ease of use, be light and wearable	
van de Laar et al., 2013 [30]				testing control settings
Vuckovic/ Osuagwu, 2013 [31]				strategies for selecting promising BCIs
Zickler et al., 2009 [105]			functionality, independence (mobility, daily life activities, employment, ease of use)	

The table below portrays the variety of different research objectives the studies were focused on. An empty box indicates that the focus of the study was not on the theme addressed in the respective column but on others

#### Qualitative studies

Only two studies assessed the opinions of potential users by means of *qualitative methods*. Klein et al. [32] set up a focus group and conducted telephone interviews with a total of 15 persons with implanted (open-loop) deep brain stimulation devices. The idea of BCI controlled closed-loop brain stimulation is met with acceptance among some potential users, while others feel ambivalent or opposed to the concept in principle. Comparing this hypothetical setting with the experiences of the study participants with open-loop brain stimulation, some participants welcomed the prospect of a self-controlled brain stimulation device while other participants maintained reservations. Some study participants expected an improved level of self-expression while others feared a distortion of their sense of self. The new brain stimulation device might also further restrain the user’s sense of accountability, in the case that others would hold the device responsible for their behavior or expressions. Furthermore, this application would require more trust in researchers to keep their data secure and confidential. This last point concerns the issue of meaningful consent. Participants mentioned difficulty in understanding all risks and implications of a closed-loop device both for themselves and for individuals with cognitive impairments.

Schicktanz, Amelung and Rieger [33] conducted open interviews with ten persons with various chronic conditions (e.g. amyotrophic lateral sclerosis (ALS), muscle atrophy, para−/tetraplegia), to whom they presented a video about a motor-readout BCI that controls a robotic arm. Participants hoped for more independence, self-control, privacy/intimacy, and better communication. Some were concerned about data protection and abuse. Additional concerns raised include the creation of self-transcending human-machine hybrids, as well as the fear for further dependencies, as the BCI necessitates service support and technological maintenance.

### Actual users

For this review, 58 studies focused on actual or real life BCI users and their experiences with the technology, usually stemming from experimental BCI studies that tested various BCI models in terms of their functionality and feasibility. The feedback from the study participants was largely limited to usability aspects. In most studies, the objective was to assess the opinion of the participants regarding the tested model and to gain information about what improvements need to be made.

#### Quantitative studies

Forty-eight studies evaluated the users' opinions or assessed various personal factors of BCI users by employing quantitative methods (mostly in the form of questionnaires). Twenty studies employed BCIs in non-impaired  individuals, 17 studies tested BCIs in individuals with various physical impairments, and ten studies included participants from both groups. Most of these studies were concerned with the usability and feasibility of the technology. The performance of BCIs for users was assessed in aspects such as efficiency, effectiveness, and satisfaction. Furthermore, popular aspects assessed included cognitive or psychological factors, such as memory, attention, fatigue, mood, motivation, depression, and quality of life. Frequently applied tests were the NASA Task Load Index (NASA-TLX) for assessing subjective workload, the Quebec User Evaluation of Satisfaction with assistive Technology, Version 2.0 (QUEST 2.0) for measuring satisfaction, the Schedule for the Evaluation of Individual Quality of Life (SeiQol) for assessing the subjective quality of life, and the Questionnaire for Current Motivation (QCM) for testing the level of motivation. Performance was measured by the accuracy of the tasks, by the number of successful trials per session, or by means of the information transfer rate (ITR). Satisfaction was assessed via QUEST 2.0, visual analog scales (VAS) or the Assistive Technology Device Predisposition Assessment (ATD-PA) scale. Thematic aspects that were covered by the quantitative studies with actual users included:

- **Usability** [34–53] in terms of ease of use/difficulty [34, 41–43, 47, 51], fatigue/exhaustion [34, 35, 39, 41], usefulness [38, 40, 43], acceptance [36, 44, 45], comfort [48, 51], or safety [44, 45].
- **Performance** [11, 36–43, 48, 49, 51, 52, 54–73] measured as accuracy [36, 41–43, 48, 52, 54, 59–62, 70], ITR [42, 48, 73], subjective level of control [58], number of successful trials [63], or skill development [67].
- **Satisfaction** [38–40, 48, 51, 56, 58, 61–66, 69–72, 74] mostly assessed via VAS, QUEST 2.0, and/or ATD-PA.
- **Psychological factors** such as motivation [38, 42, 47, 50, 55, 57, 63, 66, 75, 76], mood [11, 32, 50, 63, 66, 68], depression [38, 50, 52, 63], memory and attention [42, 44, 45], concentration [42, 77], or motor/kinesthetic imagery [68, 69, 75].
- **Workload** evaluated as efficiency via ITR and NASA-TLX [38, 40, 56, 61, 62, 71, 72], NASA-TLX only [39, 47, 53, 57, 58, 63–66, 78, 79], Repeatable Battery for Neuropsychological Status (RBANS) score [44, 45], or VAS [51].
- **Quality of life** measured via SEIQol [38, 50, 56], the Anamnestic Comparative Self-Assessment (ACSA) [38, 56] or the Psychosocial Impact of Assistive Devices Scale (PIADS) [39, 40] (some use PIADS for measuring user satisfaction, not quality of life, e.g. Vansteensel et al. [74]).
- Other aspects addressed were presence [54, 55, 70], knowledge, purpose of use, and future visions [80], plasticity and body projection [54], control/self-regulation [59, 73], comfort of (dry) electrodes [60], engagement [53], novelty [48], and physical state [11].

These aspects were assessed for non-impaired participants as well as for participants with physical impairments. Quality of life was measured among the latter only.

#### Qualitative studies

Altogether from the qualitative studies evaluating BCI users’ perspectives, 21 studies emerged. Most of the qualitative studies used interview studies or focus groups. Several also opted for observations [81, 82] or discourse analysis [33]. One study focused on non-impaired participants only [54], 16 studies on participants with physical impairments, and four studies on a mixed population. In the breakdown of mixed methods studies, there are five studies including non-impaired participants (Table 2) and 20 studies with participants with physical impairments (Table 3). The objective of such qualitative studies was to improve and develop the technology of BCI as well as to embrace a medico-technological approach. Hence, qualitative data gaining methods were used for this purpose only. The studies which did not specify any qualitative data analyzing methods mostly ran statistical analyses and addressed the qualitative data as secondary [10, 36, 38, 40, 46, 49, 54, 56, 61, 62, 71, 72, 81, 83, 84]. Certain studies aimed also to address ethical aspects related to BCIs [83, 85–87]. Şahinol [82] researched BCIs as part of a genuine sociological study including elaborated sections about the methods employed in the technology’s use. Her study describes the mutual adaptation process between human and machine, resulting in the figure of a techno-cerebral subject.

**Table 2:** Studies with non-impaired participants employing qualitative research methods

Publication	Data gaining methods	Data analyzing methods	Number of participants	Opinion towards BCI	Requests from technology	Others
Carmichael/ Carmichael, 2014 [83]	“participatory research”	none specified	10	uncertainty towards technology due to its novelty and tentative nature	more information	issues reported: cap, electrodes
Friedman et al., 2010 [54]	semi-structured interviews	none specified	10 + 3 (2 studies)			experiences of transparency of activities, sense of “presence” in VR, imagination of avatar as being (part of) themselves
Heidrich et al., 2015 [81]	participant observation	none specified	not specified	enjoyment	more efficiency	
Lightbody et al., 2010 [46]	workshops, interviews	none specified	not specified	no discomfort regarding caps	more aesthetic and practical cap, integration of other devices and entertainment system, improvements in terms of handling difficulty and graphics	
Mulvenna et al., 2012 [49]	focus groups, interviews, interactive workshops	none specified	23 + 17 (2 studies)	satisfaction, appreciation

**Table 3:** Studies with participants with physical impairments employing qualitative research methods

Publication	Data gaining methods	Data analyzing methods	Number of partici-pants	Opinion towards BCI	Issues reported	Requests from technology	Social relations	Quality of life	Personality	Future BCI scenarios
Andresen et al., 2016 [106]	interviews	thematic analysis	8			discussion limited to naming techno-logical dimensions (function, design, support) which are deemed to be of relevance	importance of social participation and communication (discussion is not directly linked to BCIs)			
Blain-Moraes et al., 2012 [90]	focus group	mix of qualitative methods (content analysis, thematic analysis)	8	offering freedom, hope, connection, independence; comfortability of learning to use the technology	mental and physical fatigue, anxiety, pain/discomfort	comfortability, ease of use, enabling communication and interlinkages to TV and phone; use in home environment; dignifying appearance	worries regarding surplus-work effort for caregivers, but also provides caregivers with more time while using BCI			
Brown et al., 2016 [84]	semistructured interviews	none specified	1 (5 inter-views)		with implant: feeling self-conscious, irritation about usage; difficulty of control	less expensive (batteries)			complexity of BCI use is at odds with the user’s “simple” and “easy-going” self-image	
Carmichael/ Carmichael, 2014 [83]	“participatory research”	none specified	8	uncertainty towards technology due to its novelty and tentative nature	cap, electrodes, frustration about BCI illiteracy	more information	participation in and contribution to research progress and technology development			
Cincotti et al., 2008 [36]	interactive discus-sions, interviews	none specified	14			home use	preference for front door opener reflects will to determine who can play a part in their social lives	raising quality of life if being used at home		
Grübler et al., 2014 [85]	semi-structured interviews	qualitative content analysis (referring to Grounded Theory Method-ology)	19	expecting physical improvement, supporting science, curiosity towards technology, overall satisfaction with BCI testing; feeling astonished about BCI control	discomfort and annoyance (prep-arations and electrodes), burden of transportation, fatigue, disappoint-ment/anger (about failure)	data security			moments of self-experience	BCIs are deemed to be impractical for everyday life use; no need for regulating BCIs
Grübler/Hildt, 2014 [87]	semi-structured interviews	(same as in Grübler et al. 2014)	19 (same as in Grübler et al. 2014)						varying opinions regarding (1) forming a functional unit with the BCI and (2) being able to forget about the technology while using it	
Heidrich et al., 2015 [81]	participant obser-vation	none specified	not specified	enjoyment		more efficiency				
Hildt, 2014 [86]	semi-structured interviews	(same as in Grübler et al. 2014)	same as in Grübler et al. 2014						varying opinions regarding (1) forming a functional unit with the BCI and (2) being able to forget about the technology while using it	
Holz, 2015 [38]	semi-structured interviews	none specified	4 + 4 + 2 (three different studies)	provides joy and happiness				provides opportunities for creativity and self-expression		
Holz et al., 2013 [56]	semi-structured interviews, focus group	none specified	4	BCIs for daily use are desirable given the technology improves	more training required	technical improve-ments, additional functions (e.g. “undo-function”)				
Holz/Botrel/ Kübler, 2015 [40]	personal statements	none specified	2	fun, happiness	increased dependence on others		participating on social public life through art exhibitions	self-esteem, expression of creativity, satis-faction		
Kübler et al., 2013 [61]	open interviews	none specified	17		set-up time, cap (comfort and look), need for washing hair after training, limited mobility, low speed					
Kübler et al., 2014 [62]	interviews	none specified	19		set-up, gel/cap, speed					ease of use and higher speed are imperatives for daily BCI use
Lightbody et al., 2010 [46]	workshop, interviews	none specified	15	satisfaction, preference for testing communi-cation functions	discontent with phone function	control of technical devices (especially TV), better ease of use	being part of research team			potential for providing more engagement and participation
Mulvenna et al., 2012 [49]	focus groups, interviews, interactive workshops	none specified	20 + 11	satisfaction, appreciation						
Şahinol, 2016 [82]	ethno-graphic field work (passive and participant obser-vations, video and audio materials, in-depth interviews)	Grounded Theory Metho-dology	6 (inter-views with study partici-pants)		physical and mental strains, frustration, belied expectations, pain		participation in studies as a pastime		on the one hand: sense of agency, cooperation with machine; on the other hand: uncertainty about causes of actions (self or machine), feeling of objectifycation due to being a study participant	
Salisbury et al., 2016 [10]	semi-structured qualitative questions	none specified	25	enjoyment						
Zickler et al., 2011 [71]	open interviews	none specified	4			control of wheelchair and other devices				daily use would require improve-ments regarding the cap, the ease of use, the size of the hardware, speed, and additional control opportunities
Zickler et al., 2013 [72]	semi-structured qualitative questions	none specified	4	enjoyment	gel induced skin problems, set-up time	improvement of the matrix, integration in other AT devices		creative expression		daily use would require less electrodes and no cable and appropriate service support

Themes that were assessed included opinions (judgements and attitudes) towards BCIs, or issues that arose during BCI testing. Others related to requests from the technology itself, such as aspects regarding social relations, quality of life, personality, and future BCI use.

Unlike non-impaired participants, persons with physical impairments have certain expectations of BCIs. Those affected by physical impairment tend to hope for more independence and social participation and expect from BCI use an increased quality of life. Some studies recognized the potential of BCI use to contribute to the user’s self-esteem and self-expression. BCIs are reported as bringing satisfaction and enjoyment, although space for improvement certainly remains in this area.

### Other BCI stakeholders

Aside from the users themselves, BCI studies also often feature inquiries into the experience of relatives, caregivers, assistants, BCI professionals, developers, health care professionals or company representatives. Expert studies consisting of BCI professionals, game developers, and therapists/health care professionals are either focused on the potential and ethical aspects of BCI (BCI professionals) or on requests and concerns regarding BCI (therapists/health care professionals) (Table 4). These external stakeholder opinions are often addressed only marginally and sometimes are not displayed separately from the users’ perspective. A recurring group of stakeholders are caregivers (and/or relatives/assistants). Their participation forms part of focus groups or compliment the user’s perspective (Table 5). Their opinions hardly are presented separately from other results.

**Table 4:** Studies with experts/professionals

Publication	Methods	Number of participants	Interest in BCI technology	Opinion towards BCI	Requests from BCI	BCI potential/future
Ahn et al., 2014 [12]	questionnaire	36 game developer, 90 researchers	developers prefer active and reactive BCIs, researchers prefer reactive BCIs	developers are more concerned about the user’s opinion in contrast to the researchers		high potential of BCI and BCI games; further potential fields: in particular rehabilitation and prosthetics
Grübler et al., 2014 [85]	survey	17 BCI professionals		ethical concerns reported: the duty of correct information transfer, avoiding unrealistic expectations in participants, BCI illiteracy, the risk of detrimental brain modifications due to BCI use and privacy issues		
Morone et al., 2015 [63]	focus group + questionnaire	15 therapists		acceptance among therapists depends on their respective technical competence and attitude; skepticism about precondition of technical knowledge/skills	future BCIs would require more goal-oriented feedback and spasticity monitoring	
Nijboer et al., 2013 [107]	survey	145 BCI professionals		disagreement regarding terminology/definitions of BCIs and marketability of different BCIs; ethical concerns reported: informed consent, benefits/risks, team responsibility, consequences, liability/personal identity, and interaction with the media; non-invasive BCIs are estimated as being of low risk (indecisive about invasive BCIs); most BCI professionals hold the view that BCI users are responsible for their actions, while being uncertain regarding issues of liability; the effect of BCI activity on personal identity and self-image on the users are deemed to be unclear		
Nijboer et al., 2014 [108]	survey + focus group	28 rehabilitation professionals (focus group: *n* = 28, survey: *n* = 18)	the professionals ascribed no added value to BCI technology		human problems and practical issues should be taken into consideration	potential BCI users are identified as those who possess intact cognition and have no extant physical or sudden movements (seizures, spasms) which can cause problems
Pedrocchi et al., 2013 [104]	focus group	14 experts (mostly health care professionals)			reproduction of natural movements, ease of use, capability of multitasking, affordability	
Zickler et al., 2011 [71]	questionnaires	3 assistive technology experts		setting too complex, setup time to long, long selection procedure, restricted mobility, prone to body movements	improved cap and gel solution	BCI as promising tool for the future

**Table 5:** Studies with caregivers/relatives

Publication	Methods	Number of participants	Points addressed
Andresen et al., 2016 [106]	qualitative interviews	7 caregivers (paid und unpaid/family caregivers)	esp. Quality of Life and AT-use emerged as major themes (results not separate from user study, see also Table 3)
Blain-Moraes et al., 2012 [90]	focus group (with users)	9 caregivers	BCI is regarded as an opportunity to maintain communication between caregivers and caretakers; caregivers would appreciate the opportunity of “back communication” (i.e. informing their caretakers, e.g. letting them know that they are on their way); caregivers also see an additional burden in dealing with the BCI (see also Table 3)
Geronimo et al., 2015 [37]	surveys (before and after testing)	41 caregivers	caregivers ranked BCI functions similar to their caretakers: priority of accuracy, variety of functions, standby reliability, wheelchair and computer control (results not separate from user study, see also Table 3)
Holz et al., 2013 [56]	focus group	3 caregivers (only featured)	focus group describes barriers for BCI use (physical, psychological, social) and its potential (freedom, independence)
Liberati et al., 2015 [91]	focus group	2 relatives + 6 caregivers and/or health professionals	reported expectations towards BCIs: information about BCIs and their applications, a system that adapts to the various stages of the disease, taking account of emotion, and retaining the users’ sense of agency

## Discussion

The systematic search presented in this review offers an outline of BCI research which employs social research methods (in all of its methodological and thematic variety.) The occurrence of empty boxes in the tabular displays above indicates the heterogeneity of the BCI studies found. Some studies focus upon and include specific aspects studied, while others concentrate on divergent aspects.

Prominent topics within BCI research have been highlighted and research gaps identified. The majority of the studies addressed are concerned with the usability and feasibility of BCIs. This provides valuable and necessary knowledge for BCI professionals to improve and optimize use of the technology. Potential users in the studies examined point out requirements they would expect of BCIs, such as standards of speed, efficiency, and ease of use. BCI use should improve their overall situation and not place an extra burden on relatives or caregivers. The general level of satisfaction that BCIs experience among actual users proves that current technological development is likely on the right track. Actual users are able to pinpoint more specific aspects which may inspire further innovation. Besides the several points addressed by potential users, there was additional mention of a more appealing design of EEG caps, a preference for dry electrodes, the integration of particular devices (e.g. TV, phone), and a stress placed on the importance of home-based use.

Apart from the conceptual disparities between the different studies mentioned above, further difficulties arise when considering the huge variety of participant groups, not only between non-impaired and impaired individuals, but also with regard to the multitude of conditions seen among the participants with physical impairments. As the majority of studies contained a mixed sample of participants with various physical impairments, specific conclusions hardly can be drawn for BCI use for particular conditions. Only a handful of studies examined persons with a singular type of impairment (often in case studies with a single participant). These impairments included stroke [57, 63], cerebral palsy [67, 81], and essential tremor [84]. The exception thus is a total of 12 studies solely (or in combination with non-impaired participants) conducted with persons having ALS [37, 39–41, 50, 58, 59, 74, 88–91]. These results do not differ significantly from the other studies. One difference might be that persons with ALS (especially those in advanced stages) show a higher degree of interest in BCIs. This may be explained by the dismal outlook for mobility from this disease, which gradually robs patients of the ability to move their limbs, communicate verbally and interact with their environment, approaching a state of locked-in syndrome. In other conditions, patients may retain certain motor functions of their body that may allow them to use other technological devices, such as peripheral neuroprostheses. Few studies have been conducted with BCI professionals.

Very little interest has been placed so far on the experiential dimension of BCI users beyond usability aspects. Where the BCI literature examines “user experience” - sometimes simply called “UX” [47] - or pursues a user-centred approach, the respective studies apply psychological factors like mood, motivation, or depression, the participants’ quality of life, satisfaction, their opinions, judgements, and requests to BCI use. In contrast to the user’s experience with BCI, the user’s experience *of* BCI has been hardly researched. This outlook would comprise questions towards BCI use from the point of view of philosophy and social sciences, broadly describable as the *What is it like?* – perspective. What is it like to use a BCI? What is it like to act by using a BCI? And therefore: What is it like to act without using my body? What is it like to be hooked up to a machine or computer? Is it (still) me that is acting within this BCI system or is it some kind of human-machine hybrid? These are questions that address topics like agency, autonomy, responsibility, accountability, self-image, identity, hybridization, or artificial intelligence.

Dealing with these questions will be of major relevance, not only because the BCI relies on a new form of connection between human and machine, but also because the technology produces bodily experiences the user would not have otherwise. For example, performing an action without moving a body part opposes our common understanding and sensation of being an agent and can manifest itself as a new experience.

First attempts in this direction have been made by Grübler and Hildt [85–87]. Their research study raised the question of transparency, i.e. the ability to operate a tool without having to consciously apply its operating instructions. Applied to the example of BCIs, this would mean operating a BCI without focusing on a mental strategy. The study in question also asked participants whether they felt themselves to be a part of a functional unit with the computer. Some participants reported experiencing transparency, but fewer participants reported having felt being a part of a functional unit with the computer. The authors postulate a discrepancy between these two judgements.

Şahinol [82] points to several aspects related to the sense of agency and shared agency with the computer, revealing uncertainties among the users about how to make sense of BCI activities. The objective of Şahinol’s study, however, is about the adaptation process between humans and machines and therefore does not elaborate on the subjective user perspective regarding these aspects. Two studies published after the completion of our data collection and review, specifically by Gilbert et al., questioned users with implanted BCIs for the treatment of epilepsy. These studies yielded valuable insights regarding control and self-perception [92] as well as autonomy and deliberation processes [93]. This step would be crucial in working to close the research gap outlined above.

Among studies with BCI professionals, the focus lies on ethics. Crucial areas of inquiry like informed consent, managing users’ expectations, and psycho-social consequences of BCI use are explored. Another recent study, conducted by Specker Sullivan et al. with neural engineers [94], was directed towards an improved sensitization of the inclusion of users in the development process of BCIs. The user-centred design endorsed in the paper is present among various studies of this review’s body of literature [38, 57, 58, 62, 72, 91]. Therapists and health care professionals are skeptical about BCIs and require certain improvements of the technology. Caregivers’ perspectives resemble users’ opinions. A study focusing on caregivers only, however, has not been conducted so far.

As most of the studies discussed in this article are guided by a medico-technological approach, ethical issues are hardly being addressed and under-researched. This observation about the field of inquiry in BCIs is discussed in detail by Specker Sullivan and Illes [95]. When ethical aspects are addressed, it is in accordance with the ethical aspects addressed in the ethics literature, as shown by Burwell et al. [96]. Matters such as autonomy, agency, personality, safety or privacy are comprised.

Our own team of researchers encourages more empirical work on these matters. While philosophical in nature, matters such as agency, autonomy and responsibility are also highly relevant for legal and policy-making affairs. This particularly depends on the part the user plays within the loop of the BCI, and its data collection. Particular closed-loop neuro devices such as closed-loop DBS modulate stimulations outside the awareness of the user and therefore cause moral and legal issues of accountability [97]. In BCIs the user receives feedback in some form (visual, auditory or haptic). She usually is nominally aware of this feedback and is given the opportunity to react to it deliberatively. The user “stays in the loop” even at least partly when connected to some autonomous system and hence gives rise to a different situation of accountability [98]. To evaluate these aspects in practice would yield important insights to inform moral, legal and political debates.

## Limitations

This study has several limitations. First of all, a scoping review cannot guarantee that all scientific literature is exhaustively found and analyzed. The literature output is confined to studies that explicitly study brain-computer- or brain-machine-interfaces or imply one of the terms as a key term. Some technologies that are labelled under “neurofeedback”, “closed-loop” or “predictive brain devices” may also qualify as brain-computer-interfaces. However, as not all of these are de facto brain-computer-interfaces and to keep the task at hand feasible, we confined our search to the search terms brain-computer* and brain-machine*. For the same reason we disregarded comparisons to other technologies such as open-loop deep brain stimulation which may render comparable results (e.g. [99]). Among the studies examined, we added manually some publications (books and book chapters) which are not peer-reviewed publications.

In order to manage the total search outcome of 73 publications, various differentiations have been neglected and need further examination: BCI varies widely in terms of its technical set-up, of which each application and model would require particular attention, e.g. in terms of measurement (invasive - non-invasive, EEG, NIRS, fMRT, ECoG, or others), mental strategy (selective attention, motor imagery), stimuli set-up (visual, auditory, haptic), application (communication program, prostheses, computer game, or others), or type of neurofeedback (displayed on monitor, successful movements, or others). Also comparisons between non-impaired participants and participants with physical impairments as well as between different impairments deserve more detailed attention. The scoping review method is a useful tool to map and synthesis large bodies of literature which comes at the expense of a detailed analysis of the results. The various themes identified in this review therefore deserve a further, elaborated examination.

## Conclusion

A great deal of research has been conducted on the perspectives of potential and actual BCI users. These opinions, and emerging social research data, are key in advancing the development of user-appropriate, humane, and successful BCI technology. BCIs have much to offer: the ability to increase quality of life, enhance social life, and contribute to a higher level of self-determination and independence for persons with physical impairments. At the same time, this technology can lead to impingements on human autonomy, psychological frustration, the creation of dependency, and the causing of confusion regarding user self-perception. The qualitative self-experience of BCI users, i.e. aspects related to personal identity, agency, and responsibility, has hardly been examined thoroughly. This is due to the delicate and demanding nature of this research as well as a limited number of study participants until now. Nonetheless, as questions regarding the experience of BCI users are crucial for evaluating ethical and societal aspects of an emerging technology, more empirical research on these matters is deeply encouraged by researchers involved.

## Additional file

Additional file 1: List of all 73 studies examining BCIs by means of social research methods. Studies are listed regarding research interest, methods, number of participants, BCI testing, BCI type, and results. (DOCX 44 kb)

## Abbreviation

BCI: Brain-computer interface

## Footnotes

1 The list in the Additional File is categorized according to research interest, methods, number of participants, BCI testing, BCI type, and results. These categories have been chosen to render a comprehensible overview of the body of literature. The purpose of the categorization is to serve as a helpful tool to the researchers, it is not part of the analyzing method applied and outlined in the review.

## References

[1] Shih JJ, Krusienski DJ, Wolpaw JR (2012). Brain-computer interfaces in medicine. Mayo Clin Proc.

[2] Brunner C, Birbaumer N, Blankertz B, Guger D, Kübler A, Mattia D, et al. BNCI Horizon 2020: towards a roadmap for the BCI community. Brain-Comput Interfaces. 2015. 10.1080/2326263X.2015.1008956 e-pub 10 Feb 2015.

[3] Bouton CE, Shaikhouni A, Annetta NV, Bockbrader MA, Friedenberg DA, Nielson DM (2016). Restoring cortical control of functional movement in a human with quadriplegia. Nature..

[4] Daly JJ, Wolpaw JR (2008). Brain–computer interfaces in neurological rehabilitation. Lancet Neurol.

[5] Graimann B, Allison B, Pfurtscheller G, Graimann B, Pfurtscheller G, Allison B (2009). Brain–Computer Interfaces: A Gentle Introduction. Brain-Computer Interfaces.

[6] Mak JN, Wolpaw JR (2009). Clinical applications of brain-computer interfaces: current state and future prospects. IEEE Rev Biomed Eng.

[7] Marchetti M, Priftis K (2015). Brain–computer interfaces in amyotrophic lateral sclerosis: a metanalysis. Clin Neurophysiol.

[8] Wolpaw JR, Birbaumer N, McFarland DJ, Pfurtscheller G, Vaughan TM (2002). Brain–computer interfaces for communication and control. Clin Neurophysiol.

[9] Chaudhary U, Birbaumer N, Ramos-Murguialday A (2016). Brain-computer interfaces for communication and rehabilitation. Nat Rev Neurol.

[10] Salisbury DB, Parsons TD, Monden KR, Trost Z, Driver SJ (2016). Brain-computer interface for individuals after spinal cord injury. Rehabil Psychol.

[11] Maksimenko VA, van Heukelum S, Makarov VV, Kelderhuis J, Lüttjohann A, Koronovskii AA (2017). Absence seizure control by a brain computer interface. Sci Rep.

[12] Ahn M, Lee M, Choi J, Jun SC (2014). A review of brain-computer interface games and an opinion survey from researchers, developers and users. Sensors (Basel, Switzerland).

[13] McFarland DJ, Daly J, Boulay C, Parvaz MA (2017). Therapeutic applications of BCI technologies. Brain-Comput Interfaces..

[14] Zafar MB, Shah KA, Malik HA. Prospects of sustainable ADHD treatment through Brain-Computer Interface systems. 2017 International Conference on Innovations in Electrical Engineering and Computational Technologies (ICIEECT). IEEE. 2017. 10.1109/ICIEECT.2017.7916532.

[15] Lebedev MA, Nicolelis MA (2006). Brain-machine interfaces: past, present and future. Trends Neurosci.

[16] Schalk G. Can electrocorticography (ECoG) support robust and powerful brain-computer interfaces? Front Neuroeng. 2010;3:9. 10.3389/fneng.2010.00009. 10.3389/fneng.2010.00009 PMC2903308 20631853

[17] Yuan H, He B (2014). Brain-computer interfaces using sensorimotor rhythms: current state and future perspectives. IEEE Trans Biomed Eng.

[18] Zander TO, Krol LR (2017). Team PhyPA: brain-computer interfacing for everyday human-computer interaction. Periodica Polytechnica Electr Eng Comput Sci.

[19] Goering S, Klein E, Dougherty DD, Widge AS (2017). Staying in the loop: relational agency and identity in next-generation DBS for psychiatry. AJOB Neurosci.

[20] Kiran AH, Verbeek P-P (2010). Trusting our selves to technology. Knowl Technol Policy.

[21] Van Den Eede Y, Rosenberger R, Verbeek P-P (2015). Tracing the tracker: A postphenomenological inquiry into self-tracking technologies. Postphenomenological investigations: Essays on human technology relations.

[22] Verbeek P-P, MH AR (2011). Subject to technology: on autonomic computing and human autonomy. Law, Human Agency, and Autonomic Computing. The Philosophy of Law meets the Philosophy of Technology.

[23] Colquhoun HLLD, O'Brien KK, Straus S, Tricco AC, Perrier L, Kastner M, Moher D (2014). Scoping reviews: time for clarity in definition, methods, and reporting. J Clin Epidemiol.

[24] Arksey H, O’Malley L (2005). Scoping studies: towards a methodological framework. Int J Soc Res Methodol.

[25] Levac D, Colquhoun H, O'Brien KK. Scoping studies: advancing the methodology. Implement Sci. 2010;5. 10.1186/1748-5908-5-69. 10.1186/1748-5908-5-69 PMC2954944 20854677

[26] Peters M, Godfrey C, Khalil H, McInerney P, Parker D, Soares CB (2015). Guidance for conducting systematic scoping reviews. Int J Evid Based Healthc.

[27] Payne G, Payne J (2004). Key concepts in social research.

[28] Moher D, Liberati A, Tetzlaff J, Altman DG (2009). The PRISMA statement. PLoS Med.

[29] Major CH, Savin-Baden M (2010). An introduction to qualitative research synthesis. Managing the inforrmation explosion in social science research.

[30] van de Laar B, Plass-Oude Bos D, Reuderink B, Poel M, Nijholt A (2013). How much control is enough? Influence of unreliable input on user experience. IEEE Transact Cybern.

[31] Vuckovic A, Osuagwu BA (2013). Using a motor imagery questionnaire to estimate the performance of a brain-computer Interface based on object oriented motor imagery. Clin Neurophysiol.

[32] Klein E, Goering S, Gagne J, Shea CV, Franklin R, Zorowitz S (2016). Brain-computer interface-based control of closed-loop brain stimulation: attitudes and ethical considerations. Brain-Comput Interfaces.

[33] Schicktanz S, Amelung T, Rieger JW (2015). Qualitative assessment of patients’ attitudes and expectations toward BCIs and implications for future technology development. Front Syst Neurosci.

[34] Allison B, Jin J, Zhang Y, Wang X (2014). A four-choice hybrid P300/SSVEP BCI for improved accuracy. Brain-Comput Interfaces..

[35] Cao T, Wan F, Wong CM, da Cruz JN, Hu Y (2014). Objective evaluation of fatigue by EEG spectral analysis in steady-state visual evoked potential-based brain-computer interfaces. Biomed Eng Online.

[36] Cincotti F, Mattia D, Aloise F, Bufalari S, Schalk G, Oriolo G (2008). Non-invasive brain-computer interface system: towards its application as assistive technology. Brain Res Bull.

[37] Geronimo A, Stephens HE, Schiff SJ, Simmons Z (2015). Acceptance of brain-computer interfaces in amyotrophic lateral sclerosis. Amyotroph Lateral Scler Frontotemporal Degen..

[38] Holz E (2015). Systematic evaluation of non-invasive brain-computer interfaces as assistive devices for persons with severe motor impairment based on a user-centred approach – in controlled settings and independent use.

[39] Holz EM, Botrel L, Kaufmann T, Kubler A (2015). Long-term independent brain-computer interface home use improves quality of life of a patient in the locked-in state: a case study. Arch Phys Med Rehabil.

[40] Holz EM, Botrel L, Kübler A (2015). Independent home use of brain painting improves quality of life of two artists in the locked-in state diagnosed with amyotrophic lateral sclerosis. Brain-Comput Interfaces..

[41] Kathner I, Kubler A, Halder S (2015). Comparison of eye tracking, electrooculography and an auditory brain-computer interface for binary communication: a case study with a participant in the locked-in state. J Neuroeng Rehabil.

[42] Kleih SC, Herweg A, Kaufmann T, Staiger-Salzer P, Gerstner N, Kubler A (2015). The WIN-speller: A new intuitive auditory brain-computer interface spelling application. Front Neurosci.

[43] Kosmyna N, Tarpin-Bernard F, Bonnefond N, Rivet B (2016). Feasibility of BCI control in a realistic smart home environment. Front Hum Neurosci.

[44] Lee TS, Goh SJ, Quek SY, Phillips R, Guan C, Cheung YB (2013). A brain-computer interface based cognitive training system for healthy elderly: a randomized control pilot study for usability and preliminary efficacy. PLoS One.

[45] Lee TS, Quek SY, Goh SJ, Phillips R, Guan C, Cheung YB (2015). A pilot randomized controlled trial using EEG-based brain-computer interface training for a Chinese-speaking group of healthy elderly. Clin Interv Aging.

[46] Lightbody G, Ware M, McCullagh P, Mulvenna MD, Thomson E, Martin S et al. A user centred approach for developing Brain-Computer Interfaces. Conference: 4th International Conference on Pervasive Computing Technologies for Healthcare, PervasiveHealth 2010, Munich, Germany, 22-25 March, 2010.

[47] Lorenz R, Pascual J, Blankertz B, Vidaurre C (2014). Towards a holistic assessment of the user experience with hybrid BCIs. J Neural Eng.

[48] Mayaud L, Cabanilles S, Van Langhenhove A, Congedo M, Barachant A, Pouplin S (2016). Brain-computer interface for the communication of acute patients: a feasibility study and a randomized controlled trial comparing performance with healthy participants and a traditional assistive device. Brain-Comput Interfaces..

[49] Mulvenna M, Lightbody G, Thomson E, McCullagh P, Ware M, Martin S (2012). Realistic expectations with brain computer interfaces. J Assist Technol.

[50] Nijboer F, Birbaumer N, Kubler A. The influence of psychological state and motivation on brain-computer interface performance in patients with amyotrophic lateral sclerosis - a longitudinal study. Front Neurosci. 2010;4. 10.3389/fnins.2010.00055. 10.3389/fnins.2010.00055 PMC2916671 20700521

[51] Peters B, Mooney A, Oken B, Fried-Oken M (2016). Soliciting BCI user experience feedback from people with severe speech and physical impairments. Brain-Comput Interfaces.

[52] Poletti B, Carelli L, Solca F, Lafronza A, Pedroli E, Faini A (2016). Cognitive assessment in amyotrophic lateral sclerosis by means of P300-brain computer Interface: a preliminary study. Amyotroph Lateral Scler Frontotemporal Degen.

[53] Gürkök H, Hakvoort G, Poel M, Anacleto JC, Fels S, Graham N, Kapralos B, Saif El-Nasr M, Stanley K (2011). Evaluating User Experience in a Selection Based Brain-Computer Interface Game A Comparative Study. Entertainment Computing – ICEC 2011: 10th International Conference, ICEC 2011, Vancouver, Canada, October 5–8, 2011. Proceedings.

[54] Friedman D, Leeb R, Pfurtscheller G, Slater M (2010). Human-computer Interface issues in controlling virtual reality with brain-computer Interface. Human-Comput Interaction.

[55] Friedrich EVC, Scherer R, Neuper C (2013). Long-term evaluation of a 4-class imagery-based brain–computer interface. Clinical Neurophysiology..

[56] Holz EM, Hohne J, Staiger-Salzer P, Tangermann M, Kubler A (2013). Brain-computer interface controlled gaming: evaluation of usability by severely motor restricted end-users. Artif Intell Med.

[57] Schreuder M, Riccio A, Risetti M, Dahne S, Ramsay A, Williamson J (2013). User-centered design in brain-computer interfaces-a case study. Artif Intell Med.

[58] Botrel L, Holz EM, Kübler A (2015). Brain painting V2: evaluation of P300-based brain-computer interface for creative expression by an end-user following the user-centered design. Brain-Comput Interfaces..

[59] Grosse-Wentrup M, Schölkopf B (2014). A brain-computer interface based on self-regulation of gamma-oscillations in the superior parietal cortex. J Neural Eng.

[60] Guger C, Krausz G, Allison BZ, Edlinger G. Comparison of dry and gel based electrodes for P300 brain-computer interfaces. Front Neurosci. 2012:60. 10.3389/fnins.2012.00060. 10.3389/fnins.2012.00060 PMC3345570 22586362

[61] Kübler A, Holz E, Kaufmann T, Zickler C. A User Centred Approach for Bringing BCI Controlled Applications to End-Users. 2013. 10.5772/55802.

[62] Kubler A, Holz EM, Riccio A, Zickler C, Kaufmann T, Kleih SC (2014). The user-centered design as novel perspective for evaluating the usability of BCI-controlled applications. PLoS One.

[63] Morone G, Pisotta I, Pichiorri F, Kleih S, Paolucci S, Molinari M (2015). Proof of principle of a brain-computer interface approach to support poststroke arm rehabilitation in hospitalized patients: design, acceptability, and usability. Arch Phys Med Rehabil.

[64] Riccio A, Leotta F, Bianchi L, Aloise F, Zickler C, Hoogerwerf EJ (2011). Workload measurement in a communication application operated through a P300-based brain-computer interface. J Neural Eng.

[65] Riccio A, Holz EM, Arico P, Leotta F, Aloise F, Desideri L (2015). Hybrid P300-based brain-computer interface to improve usability for people with severe motor disability: electromyographic signals for error correction during a spelling task. Arch Phys Med Rehabil.

[66] Rohm M, Schneiders M, Muller C, Kreilinger A, Kaiser V, Muller-Putz GR (2013). Hybrid brain-computer interfaces and hybrid neuroprostheses for restoration of upper limb functions in individuals with high-level spinal cord injury. Artif Intell Med.

[67] Taherian S, Selitskiy D, Pau J, Davies TC, Owens RG (2016). Training to use a commercial brain-computer interface as access technology: a case study. Disabil Rehabil Assist Technol.

[68] Vasilyev A, Liburkina S, Yakovlev L, Perepelkina O, Kaplan A (2017). Assessing motor imagery in brain-computer interface training: psychological and neurophysiological correlates. Neuropsychologia..

[69] Vourvopoulos A, Bermudezi BS (2016). Motor priming in virtual reality can augment motor-imagery training efficacy in restorative brain-computer interaction: A within-subject analysis. J neuroeng Rehabil.

[70] Won DO, Hwang HJ, Dahne S, Muller KR, Lee SW (2016). Effect of higher frequency on the classification of steady-state visual evoked potentials. J Neural Eng.

[71] Zickler C, Riccio A, Leotta F, Hillian-Tress S, Halder S, Holz E (2011). A brain-computer interface as input channel for a standard assistive technology software. Clin EEG Neurosci.

[72] Zickler C, Halder S, Kleih SC, Herbert C, Kubler A (2013). Brain painting: usability testing according to the user-centered design in end users with severe motor paralysis. Artif Intell Med.

[73] van de Laar B, Reuderink B, Bos DPO, Heylen D (2010). Evaluating user experience of actual and imagined movement in BCI gaming. Int J Gaming Computer-Mediated Simul.

[74] Vansteensel MJ, Pels EG, Bleichner MG, Branco MP, Denison T, Freudenburg ZV (2016). Fully implanted brain-computer Interface in a locked-in patient with ALS. N Engl J Med.

[75] Hammer EM, Halder S, Blankertz B, Sannelli C, Dickhaus T, Kleih S (2012). Psychological predictors of SMR-BCI performance. Biol Psychol.

[76] Kleih SC, Nijboer F, Halder S, Kübler A (2010). Motivation modulates the P300 amplitude during brain-computer interface use. Clin Neurophysiol.

[77] da Silva-Sauer L, Valero-Aguayo L, de la Torre-Luque A, Ron-Angevin R, Varona-Moya S (2016). Concentration on performance with P300-based BCI systems: a matter of interface features. Appl Ergon.

[78] Felton EA, Williams JC, Vanderheiden GC, Radwin RG (2012). Mental workload during brain-computer interface training. Ergonomics..

[79] Hortal E, Ubeda A, Ianez E, Azorin JM (2014). Control of a 2 DoF robot using a brain-machine interface. Comput Methods Prog Biomed.

[80] Cloyd TD (2014). (r)evolution in brain-computer Interface Technologies for Play: (non)users in mind.

[81] Heidrich RO, Jensen E, Rebelo F, Oliveira T (2015). A comparative study: use of a brain-computer Interface (BCI) device by people with cerebral palsy in interaction with computers. Anais da Academia Brasileira de Ciencias.

[82] Şahinol M (2016). Das techno-zerebrale Subjekt: Zur Symbiose von Mensch und Maschine in den Neurowissenschaften.

[83] Carmichael C, Carmichael P (2014). BNCI systems as a potential assistive technology: ethical issues and participatory research in the BrainAble project. Disabil Rehabil Assist Technol..

[84] Brown T, Thompson MC, Herron J, Ko A, Chizeck H, Goering S (2016). Controlling our brains – a case study on the implications of brain-computer interface-triggered deep brain stimulation for essential tremor. Brain-Comput Interfaces..

[85] Grübler G, Al-Khodairy A, Leeb R, Pisotta I, Riccio A, Rohm M (2014). Psychosocial and ethical aspects in non-invasive EEG-based BCI research—a survey among BCI users and BCI professionals. Neuroethics..

[86] Hildt E, Friedrich O, Michael Z (2014). Interaktion per Gehirn mit einem Computer. Persönlichkeit: Neurowissenschaftliche und neurophilosophische Fragestellungen.

[87] Grübler G, Hildt E (2014). Brain-computer-interfaces in their ethical, social and cultural contexts. The international library of ethics, law and technology.

[88] Huggins JE, Wren PA, Gruis KL (2011). What would brain-computer interface users want? Opinions and priorities of potential users with amyotrophic lateral sclerosis. Amyotroph Lateral Scler.

[89] Kageyama Y, Hirata M, Yanagisawa T, Shimokawa T, Sawada J, Morris S (2014). Severely affected ALS patients have broad and high expectations for brain-machine interfaces. Amyotroph Lateral Scler Frontotemporal Degener.

[90] Blain-Moraes S, Schaff R, Gruis KL, Huggins JE, Wren PA (2012). Barriers to and mediators of brain-computer interface user acceptance: focus group findings. Ergonomics..

[91] Liberati G, Pizzimenti A, Simione L, Riccio A, Schettini F, Inghilleri M (2015). Developing brain-computer interfaces from a user-centered perspective: assessing the needs of persons with amyotrophic lateral sclerosis, caregivers, and professionals. Appl Ergon.

[92] Gilbert F, Cook M, O'Brien T, Illes J. Embodiment and estrangement: results from a first-in-human “intelligent BCI” trial. Sci Eng Ethics. 2017. 10.1007/s11948-017-0001-5. 10.1007/s11948-017-0001-5 PMC6418065 29129011

[93] Gilbert F, O'Brien T, Cook M (2018). The effects of closed-loop brain implants on autonomy and deliberation: what are the risks of being kept in the loop?. Camb Q Healthc Ethics.

[94] Specker Sullivan L, Klein E, Brown T, Sample M, Pham M, Tubig P (2018). Keeping disability in mind: a case study in implantable brain–computer Interface research. Sci Eng Ethics.

[95] Specker Sullivan L, Illes J (2018). Ethics in published brain-computer interface research. J Neural Eng.

[96] Burwell S, Sample M, Racine E (2017). Ethical aspects of brain computer interfaces: a scoping review. BMC Med Ethics.

[97] Glannon W, Ineichen C, El Hady A (2016). Philosophical aspects of closed-loop neuroscience. Closed loop neuroscience.

[98] Kellmeyer P, Cochrane T, Mueller O, Mitchell C, Ball T, Fins JJ (2016). The effects of closed-loop medical devices on the autonomy and accountability of persons and systems. Camb Q Healthc Ethics.

[99] Gilbert F, Goddard E, Viaña JNM, Carter A, Horne M (2017). I miss being me: phenomenological effects of deep brain stimulation. Am J Bioeth Neurosci.

[100] Blabe CH, Gilja V, Chestek CA, Shenoy KV, Anderson KD, Henderson JM (2015). Assessment of brain-machine interfaces from the perspective of people with paralysis. J Neural Eng.

[101] Collinger JL, Boninger ML, Bruns TM, Curley K, Wang W, Weber DJ (2013). Functional priorities, assistive technology, and brain-computer interfaces after spinal cord injury. J Rehabil Res Dev.

[102] Huggins JE, Moinuddin AA, Chiodo AE, Wren PA (2015). What would brain-computer interface users want: opinions and priorities of potential users with spinal cord injury. Arch Phys Med Rehab.

[103] Lahr J, Schwartz C, Heimbach B, Aertsen A, Rickert J, Ball T (2015). Invasive brain-machine interfaces: a survey of paralyzed patients’ attitudes, knowledge and methods of information retrieval. J Neural Eng.

[104] Pedrocchi A, Ferrante S, Ambrosini E, Gandolla M, Casellato C, Schauer T (2013). MUNDUS project: MUltimodal neuroprosthesis for daily upper limb support. J Neuroeng Rehabil..

[105] Zickler C, Di Donna V, Kaiser V, Al-Khodairy A, Kleih S, Kübler A, Hoogerwerf EJ (2009). BCI Applications for People with Disabilities: Defining User Needs and User Requirements. 10th European Conference for the Advancement of Assistive Technology.

[106] Andresen EM, Fried-Oken M, Peters B, Patrick DL (2016). Initial constructs for patient-centered outcome measures to evaluate brain-computer interfaces. Disabil Rehabil Assist Technol..

[107] Nijboer F, Clausen J, Allison BZ, Haselager P (2013). The Asilomar survey: Stakeholders’ opinions on ethical issues related to brain-computer interfacing. Neuroethics..

[108] Nijboer F, Plass-Oude Bos D, Blokland Y, van Wijk R, Farquhar J (2014). Design requirements and potential target users for brain-computer interfaces – recommendations from rehabilitation professionals. Brain-Comput Interfaces..

