# Supplementary material for: Using brain-computer interfaces: a scoping review of studies employing social research methods
Source: BMC Med Ethics. 2019 Mar 7;20:18. doi: 10.1186/s12910-019-0354-1 (PMC6407281; doi:10.1186/s12910-019-0354-1)
Supplement: Supplementary file 1 — List of all 73 studies examining BCIs by means of social research methods. Studies are listed regarding research interest, methods, number of participants, BCI testing, BCI type, and results. (DOCX 44 kb) [file 12910_2019_354_MOESM1_ESM.docx]

**Additional file 1. List of all 73 studies examining BCIs by means of social research methods.**

| **Publication** | **Subject/Research Interest** | **Method** | | **n (number of participants)** | **Actual BCI Testing/Experience** | **BCI type** | **Results/Findings** |
| --- | --- | --- | --- | --- | --- | --- | --- |
| Ahn et al., 2014 | collecting opinions of BCI gaming stakeholders | quant.: questionnaire | | 294 non-impaired participants (168 users, 36 game developer, 90 researchers) | no |  | BCIs and BCI games are perceived to have a high influence among applied technologies; according to the participants, the most promising applications are rehabilitation and prosthesis followed by gaming; users and developers prefer active and reactive BCIs, researchers prefer reactive BCI; developers are more concerned about the user's opinion than researchers |
| Allison et al., 2014 | evaluation of hybrid BCIs | quant.: questionnaire | | 10 non-impaired participants | yes (3 different settings, number of sessions = 1(?)) | P300, SSVEP and P300/SSVEP-hybrid BCI | the hybrid BCI yields similar results in terms of subjective experience as the P300 and the SSVEP applications, the numbers however display a higher level in annoyance, difficulty and fatigue for the hybrid in comparison to the SSVEP and the P300 (except: lower level for annoyance in comparison to the P300) |
| Andresen et al., 2016 | evaluation of BCIs via "patient-centred outcome measures" | quant.: questionnaires (incl. NASA-TLX, MQE, PIADS, QUEST, QUIS, FuNHRQOL)  qual.: interviews | | 8 participants with severe speech and physical impairments + 7 caregivers | participants with impairments: yes (1 - 7 sessions) caregivers: no | RSVP-keyboard BCI | the ICF framework turned out as being sufficient for mapping items related to body functions but insufficient in terms of personal factors; Quality of Life and AT-use emerged as major themes |
| Blabe et al., 2015 | testing 8 BMI technologies | quant.: survey | 156 participants with cervical spinal cord injuries | | no |  | high priority has been given to restoring upper extremity function, esp. high-performance typing; this shows the priority of communication over wheelchair steering or robotic devices; BMI are unappealing when difficult to use or aesthetically unpleasing; appearance plays an important role: many rather would opt for an implant than for an EEG cap ( despite concerns regarding surgery); reported requirements for the technology to be adopted: autonomous, unobtrusive, no/little maintenance, high performing |
| Blain-Moraes et al., 2012 | analysing participants’ acceptance of BCIs | qual.: focus group | | 8 participants with ALS + 9 caregivers | participants with ALS: yes (2-3 sessions) caregivers: no | P 300 BCI speller | participants regard “relational factors” such as “corporeal, technological and social relations with the BCI” higher than “personal factors”, i.e. “physical, physiological and psychological concerns”; participants see a lot of “opportunities” within the technology, even offering “freedom” and “hope”; concerns towards the practicability of BCIs were: the tiring handling of BCIs, cognitive fatigue, discomfort of the cap, distractions and anxiety in relation to the requested concentration level, and suspicion towards technology in general; the main expectation towards the technology consists of the provision of communication channels; BCIs provide the possibility of maintaining communication, but at the same time increase the caregivers’ workload |
| Botrel et al., 2015 | evaluation of Brain Painting V2 | quant.: NASA-TLX questionnaire, (informal) interview | | 1 (BCI experienced) participant with ALS, 1 caregiver/assistant (being interviewed) | yes (33 sessions) | P300-based BCI (BP2) | BP2 is reported to be easier to handle than BP1 (assistant); BP2 doesn't require more effort than BP1; (home-based) BCI is being used with moderate to high satisfaction |
| Brown et al., 2016 | evaluation of BCI-mediated deep brain stimulation | qual.: interviews (5 semistructured interviews) | | 1 participant with essential tremor | yes (number of sessions not specified) | BCI-DBS (implanted neurostimulator) | the participant reported self-consciousness about and permanent awareness of the implant, difficulty in acclimating to the implant, uncertainty about its use, difficulty in gaining BCI control; discrepancy between self-image (being "simple-minded", "easy-going" and having a restless mind) and BCI-control |
| Cao et al., 2014 | testing fatigue while using a SSVEP-BCI | quant.: questionnaire (before and after the task) | | 21 non-impaired participants (students) | yes (2 sessions) | SSVEP-BCI | an increasing level of fatigue can be displayed during SSVEP-BCI training |
| Carmichael/Carmichael, 2014 | assessing ethical aspects in BCI research | unknown ("participatory research") | | 8 participants with physical impairments + 10 non-impaired participants | yes (3 sessions) | BrainAble (hybrid system that uses different BCI signals) | participants reported concerns regarding the electrode-laden EEG cap and its aesthetics and the gel electrodes; the prototypical nature of the BrainAble-system and BCI illiteracy proved to be further issues |
| Cincotti et al., 2008 | testing BCIs for house-environment | quant.: questionnaire  qual.: interactive discussions (participants + their relatives) + interviews (participants) | | 14 non-impaired participants + 14 participants with severe motor disabilities (4 of them operated a BCI) | yes (5-12 sessions) | BCI2000 | BCI performance scored lower than the muscle-based input application; participants expect more privacy and quality of life from successful implementations of these home-environment systems; accordingly the front door opener was reported as their favourite output device |
| Cloyd, 2014 | examining BCI technologies for gaming and consumer applications | quant.: user survey qual.: interview | | 72 BCI users (online questionnaire) + 1 telephone interview (representative from NeuroSky) | yes (level of BCI experience not assessed) | focus: NeuroSky and Emotiv BCIs | BCI technology is deemed to be based on contextual factors such as economic, socio-cultural, and material aspects; the success of BCI’s, however, relies, according to the author, on the users' acceptance and interpretation |
| Collinger et al., 2013 | assessing functional priorities, technical knowledge, and BCI preferences | quant.: survey | | 57 participants (veterans) with spinal cord injuries | no |  | arm/hand and bladder/bowel function were prioritized over walking function; most participants would use a BCI in case it doesn't cause too much inconvenience; there is a clear priority in seeing BCIs as helpful in working with FES, less priority for controlling, robotic devices, computers or wheelchairs via BCI; most important BCI design characteristic: independent operation |
| da Silva-Sauer et al., 2016 | testing the effect of concentration on BCI performance | quant.: questionnaire | | 14 non-impaired participants | yes (3 sessions) | P300-BCI speller | adding dynamic applications like a T9 speller allows for a lower concentration level; the less dynamic the interface the more concentration is needed |
| Felton et al., 2012 | evaluation of subjective mental work load during BCI-training between non-impaired and impaired users | quant.: survey (NASA Task Load Index) | | 12 non-impaired participants + 7 participants with physical impairments | yes ( 6 or more sessions) | BCI2000 | the mental workload during BCI-training is about the same for non-impaired and users with physical impairments |
| Friedman et al., 2010 | testing BCIs in virtual reality systems | quant.: questionnaires qual.: semistructured interview | | 10 (experiment 1) + 3 non-impaired participants (experiment 2) | yes (1-3 sessions) | VR-BCI | experiment 1: participants performed better when given clear instructions in comparison to a free-choice task; experiment 2: controlling a virtual body via BCI has proven to be feasible; participants even showed signs of transparency |
| Friedrich et al., 2013 | evaluation of a 4-class imagery-based BCI | quant.: questionnaires | | 14 non-impaired participants | yes (10 sessions + 1 follow-up session; 12 of 14 participants) | EEG-based 4-class BCI | the 4-class (4 different mental tasks) BCI proved to be feasible; ease of use and quality of imagery increased with the number of training sessions while their fear of inability decreased |
| Geronimo et al., 2015 | assessing acceptance of BCIs among ALS participants | quant.: surveys (before and after testing) | | 42 participants with ALS + 41 caregivers | participants with ALS: yes (4 BCI sessions) caregivers: no | P300 speller + motor-imagery cursor control | highest ranked system features: accuracy, variety of functions, and standby reliability; priority of wheelchair and computer control; participants with cognitive impairments displayed a higher interest in using BCIs while participants with behavioural impairments were less receptive of BCIs |
| Grosse-Wentrup/ Schölkopf, 2014 | testing a BCI based on self-regulation | quant.: questionnaire | | 19 non-impaired participants + 1 person with ALS (no questionnaire) | yes (1 session, 3 participants had 5 sessions) | SMR-based BCI (EEG) | mental strategy of alternating between states of focused attention and relaxation is feasible and can be used as an alternative BCI paradigm |
| Grübler et al., 2014 | analyzing ethical, legal, and social issues concerning BCIs | quant.: survey qual.: semistructured interviews | | 19 participants with motor impairments + 17 BCI professionals | participants: yes (number of sessions not specified) professionals: no | EEG-based non-invasive BCIs | participants expressed discomfort due to the extensive preparation procedure, the EEG cap including wet electrodes, issues regarding BCI control and system security; additionally, BCI professionals reported several fears: the duty of correct information transfer, avoiding unrealistic expectations in participants, BCI illiteracy, the risk of detrimental brain modifications due to BCI use and privacy issues |
| Grübler/Hildt, 2014 | collection about ethical, social and legal issues concerning BCI including different stakeholder | qual.: semistructured interviews (as in Grübler et al. 2014) | | 19 participants with motor impairments (as in Grübler et al. 2014) | yes (number of sessions not specified) | EEG-based non-invasive BCIs | results as in Grübler et al. 2014 and Hildt 2014 |
| Guger et al., 2012 | testing dry electrodes on BCIs | quant.: questionnaire | | 23 non-impaired participants | yes (1 session) | P300-based BCI speller | no discomfort has been reported regarding the dry electrodes |
| Gürkok et al., 2011 | evaluation of user-experience in a selection-based BCI game | quant.: questionnaires (NASA-TLX, GEQ, AttrakDiff2) | | 20 non-impaired participants | yes (1 session) | SSVEP-based BCI | the users prefer an automatic speech recognizer (ASR) control over the BCI control in terms of workload, usability and engagement |
| Hammer et al., 2011 | investigating psychological parameters of BCI performance | quant.: questionnaire (Vividness of movement imagery questionnaire) + performance, personality and clinical tests | | 83 non-impaired BCI novices | yes (1 session) | "Berlin-BCI" (SMR-controlled BCI) | psychological parameters such as attention span, personality, or motivation, have a decisive effect on BCI performance |
| Heidrich et al., 2015 | product evaluation of NeuroSky MindWave: comparing non-impaired user and participants with cerebral palsy | qual.: case study (participant observation) | | participants with cerebral palsy + non-impaired users (partly students); partly children (even age of 3) (number of sessions not specified) | yes (testing 4 games) | NeuroSky MindWave | no differences between non-impaired user and participants with cerebral palsy |
| Hildt, 2014 | evaluation of philosophical/ethical implications of BCI-use | qual.: semistructured interviews | | 20 participants (7 with stroke, 13 with motor impairments) | yes (number of sessions not specified) | unknown | four participants reported a sense of functional unit with technical system, 15 participants reported a sense of "transparency" while operating a BCI |
| Holz, 2015 | systematic evaluation of BCIs based on a user-centred approach (3 different studies/contexts) | quant.: questionnaires qual.: semistructured interviews | | 3 studies: 4 participants with severe motor impairment; 10 non-impaired participants + 4 participants with severe motor impairments; 2 participants with ALS | yes (9 sessions; 3 sessions; 352 + 158 sessions) | motor-imagery based BCI; hybrid P300 ERP based BCI; Brain Painting | BCIs improve the quality life of end-users in terms of happiness, usefulness, self-confidence, self-esteem, productivity, well-being and participation; BCIs at the same time reduces frustration and confusion levels, but partly also reduces levels of independence due to being dependent on assistance for setting up the system |
| Holz et al., 2013 | testing BCI gaming | quant.: questionnaires (NASA-TLX, VAS satisfaction, Extended QUEST 2.0, ATD-PA)  qual.: semistructured interviews + focus group | | 4 participants with severe motor impairments + caregivers (in focus group) | participants: yes (6 sessions) caregivers: no | SMR-BCI gaming application Connect-Four | participants reported satisfaction regarding weight, safety, professional services and learnability, moderate satisfaction with comfort and aesthetics, and dissatisfaction with cap/electrodes, adjustment, ease of use, effectiveness, reliability, and speed; different BCIs are suited for different individuals; users are more tolerant towards malfunctioning in BCIs when they are not meant for communication purposes |
| Holz et al., 2015 | testing BCI home use (in terms of efficiency, effectiveness, satisfaction, and quality of life) | quant.: case study (questionnaires) | | 1 participant with ALS (Locked-In State) | yes (200 sessions) | visual P300 BCI (Brain Painting) | BCI use improved the patient's quality of life (in terms of competence, adaptability, self-esteem), but had a negative impact on independence (dependence on caregivers); reasons for dissatisfaction were technical problems and varying BCI control |
| Holz/Botrel/  Kübler, 2015 | evaluation of home-based BCI | quant.: questionnaires (NASA-TLX, QUEST 2.0, PIADS, ATD-PA, VAS)  (qual.: personal statements) | | 2 participants with ALS | yes (152 + 158 sessions) | Brain Painting | Brain Painting has a positive effect on self-esteem, competence and adaptability, increases the quality of life of the participants, but also increases their dependence on others (for setting up the BCI) |
| Hortal et al., 2014 | testing two strategies for controlling BCI mediated robot | quant.: questionnaire (NASA TLX) | | 4 (non-impaired(?)) participants | yes (2 sessions) | SVM-BCI | the hierarchical control strategy is regarded to require a higher workload than the directional control strategy |
| Huggins et al., 2011 | evaluation of BCIs among participants with ALS | quant.: survey (via telephone) | | 61 participants with ALS | no |  | participants showed a strong interest in using a BCI; a majority (72 %) is even willing to undergo surgery in order to operate with an invasive BCI; functional preferences of the BCIs reported by the participants (i.e. accuracy, speed, simplicity, and standby mode reliability) cannot be matched by current BCIs |
| Huggins et al., 2015 | evaluation of BCIs among participants with spinal cord injuries | quant.: survey (via telephone and face-to-face) | | 40 participants (30 potential users, 10 BCI study participants, being questioned before their first BCI training session) | no |  | participants being regarded as having low functional independence are very interested in BCIs; BCIs need to improve their speed and setup time in order to match the participants’ expectations |
| Kageyama et al., 2014 | evaluation of BCIs among participants with (severe) ALS | quant.: questionnaire | | 37 participants with ALS | no |  | there is interest among participants with ALS in BCIs in order to improve their communication capabilities; the severity of the disease and the usage of a PC have positive impacts on their interest in BCIs |
| Käthner et al., 2015 | comparison of different ATs (incl. auditory BCI) | quant.: case study (questionnaires) | | 1 participant with ALS (Locked-In State) | yes (3 sessions) | auditory ERP-BCI | the BCI was rated among the tested technologies as the one which is the easiest to use and as the most tiring one; the BCI might become interesting for the participant once his eye muscle will not be functional anymore |
| Kleih et al., 2010 | examination of motivation as an effect on BCI performance | quant.: questionnaire | | 33 non-impaired students | yes (number of sessions =1(?)) | ERP-BCI (P300-amplitude) | motivation proved to have an effect on BCI performance: motivated participants were able to perform faster than less motivated participants; the participants' motivation were not affected by monetary reward; high motivation may be explained by high performance, both may be ascribed to a high level of interest |
| Kleih et al., 2015 | evaluation of the WIN-speller | quant.: questionnaire with non-impaired participants, interviews with participants with motor impairments | | 11 non-impaired participants + 4 participants with motor impairments | yes (participants with motor impairments: 1 session, non-impaired participants: 2 sessions) | auditory BCI (WIN-speller) | WIN-speller is reported to be more difficult but easier to focus attention on as compared to a visual or multimodal BCI paradigm (non-impaired users); users showed surprise regarding the efficiency of the BCI, but wished for a faster presentation of the stimuli; the WIN-speller renders no significant correlations with attention, memory, self-efficacy belief, and behavioural orientation |
| Klein et al., 2016 | evaluation of ethical issues among (implanted) BCI-controlled DBS users | qual.: focus group, telephone interviews | | 15 participants with implanted DBS (focus group: n=8, interviews: n=7) | no |  | regarding closed looped DBS systems (which would be BCI operated) four major themes are presented as relevant (control, authentic self, relationship effects, and meaningful consent): the participants hold ambivalent views on all four aspects |
| Kosmyna et al., 2016 | evaluation of BCI controlled Smart Home | quant.: USE questionnaire | | 12 non-impaired participants (with BCI experience) + 2 participants with motor impairments (with BCI experience) | yes (1 session) | EEG BCI (Domus Smart Home) | participants with motor impairments display a higher level of motivation as they have to gain more from successful BCI use in comparison to non-impaired participants; the participants ascribed a high level regarding each usefulness, satisfaction, ease of use and learning to the BCI; participants with motor impairments performed better than non-impaired participants, explanations given: motivation and less interference from muscle movement |
| Kübler et al., 2013 | applying user-centred design to 4 BCI-applications (home environment) | quant.: questionnaires qual.: open interviews | | 17 participants with physical impairments (including participants in the locked-in state) | yes (4-8 sessions) | P300, EMG & SMR-BCI | measured variables were effectiveness, efficiency and satisfaction; effectiveness varied between P300 and SMR controlled use (high for P300; low for SMR); efficiency and satisfaction were moderate; satisfaction was high regarding independent home use, even when the performance level was classified as moderate |
| Kübler et al., 2014 | evaluation of different BCI applications | quant.: questionnaires qual.: interviews | | 19 participants with motor impairments | yes (1-7 sessions) | P300, ERP- and SMR-BCI (Brain Painting, Spelling, Spelling-hybrid, Connect-Four) | participants were more satisfied with BCIs that are designed for entertainment purposes (Brain Painting, Connect 4); BCIs for communication purposes (even when tested better in terms of efficiency, effectiveness or reliability) don't meet the users’ needs |
| Lahr et al., 2015 | evaluation of invasive BMIs | quant.: survey (questionnaire) | | 131 paralyzed participants | no |  | most participants had knowledge of invasive BMIs; most are open to invasive BMIs (esp. ALS participants) but acceptance will rely on the risk-reward ratio |
| Lee et al., 2013 | evaluation of BCIs among elderly people (regarding the improvement of memory and attention) | quant.: questionnaires | | 31 non-impaired elderly (English speaking) | yes (24 sessions) | BCI Based Memory and Attention Training Game (EEG) | positive feedback in terms of usability and acceptability, high motivation; BCI training shows a moderate positive effect on attention and memory |
| Lee at al., 2015 | evaluation of BCIs among elderly people (regarding the improvement of memory and attention) | quant.: questionnaires | | 31 non-impaired elderly (Chinese speaking) | yes (24 sessions) | BCI Based Memory and Attention Training Game (EEG) | same results as in Lee et al., 2013; only difference: in contrast to the English speaking cohort, the participants of this study reported various adverse events |
| Liberati et al., 2015 | assessing needs of potential BCI usage among ALS stakeholders | qual.: focus group | | 1 participant with ALS + 2 relatives + 6 caregivers and/or health professionals | no |  | reported expectations towards BCIs: information about BCIs and their applications, a system that adapts to the various stages of the disease, taking account of emotion by the technology, and retaining the user’s sense of agency |
| Lightbody et al., 2010 | testing a user-centred approach for developing BCIs | quant.: questionnaire qual.: workshops and interviews | | 15 participants with physical impairments + non-impaired participants (number of sessions not specified) | yes (6 sessions) | SSVEP, P300 and ERD/ERS-BCIs (3 paradigms) | the use of a EEG cap including electrodes didn't cause significant discomfort to the participants; participants’ assessments reveal that the prime function of the BCI-system is communication (by means of multimedia content); especially television shows to be most important and participants wish for future BCI software to be integrated into television |
| Lorenz et al., 2014 | assessing the user experience ("holistic assessment") of hybrid BCIs | quant.: questionnaires (NASA-TLX, UX questionnaire) | | 12 non-impaired participants | yes (1 sessions) | BCI-driven interfaces (ERP, MI, GUI) | so-called “hedonic aspects” were in line with “pragmatic aspects”, for instance accuracy and speed; the more efficient the BCI the more enjoyable it appears to be for its users |
| Mayaud et al., 2016 | evaluation/comparison of different BCIs and among different study groups | quant.: VAS questionnaire | | 12 participants with quadriplegia (study 1) + 10 participants with quadriplegia + 9 non-impaired participants (study 2) | yes (2 x 3 sessions) | P300 BCI speller + RoBIK BCI | traditional ATs were tested as being superior to BCIs due to the level of fatigue BCIs are causing; performance wise there is no difference between participants with physical impairments and non-impaired users, however, the former reported a higher level of fatigue |
| Morone et al., 2015 | evaluation of BCI-assisted training in stroke rehabilitation | quant.: questionnaires (participants) qual.: focus group (therapists) | | 8 participants with stroke (arm plegia or paresis) + 15 therapists | participants: yes (12 sessions) therapists: no | motor imagery based BCI (EEG), BCI2000 software | positive correlations were found between satisfaction and motivation as well as between BCI performance on the one side and interest and motivation on the other; acceptance among therapists depends on their respective technical competence and attitude |
| Mulvenna et al., 2012 | evaluating BCIs for home-based use | quant.: surveys qual.: focus groups + interviews + "interactive workshops" | | first 5, then 20 participants with impairments; 11 participants with impairments + 17 non-impaired participants; 23 non-impaired participants | yes (number of sessions unknown) | SSVEP-BCI | the BCI tested better among non-impaired users compared to users with impairments, therefore BCIs need to be improved |
| Nijboer et al., 2010 | examination of the impact of motivational factors and well-being on BCI performance among ALS participants | quant.: questionnaires | | 7 participants with ALS | yes (30 sessions) | SMR-BCI and P300-BCI | according to the results, mood has no significant impact on BCI performance; among individual participants correlations between motivation (positively), depression and incompetence fear (negatively) occurred; comparing the two BCI paradigms, it can be stated that the P300 has a higher information transfer rate while motivation and mood are more often related to the number of BCI sessions with SMR-BCI compared to sessions with a P300-BCI |
| Nijboer et al., 2013 | assessment of BCI-professionals' opinion on terminology, marketing potential and ethics | quant.: survey (at an international BCI conference) | | 145 BCI professionals | not assessed |  | the study reports a tremendous amount of disagreement among the professional community regarding terminology and definitions of BCIs; also considering the marketability of different BCIs, different views prevailed; the survey contained following ethical issues: informed consent, benefits/risks, team responsibility, consequences, liability/personal identity, and interaction with the media; the majority of the BCI community believes in the benefits and low risk of non-invasive BCIs, but is indecisive in terms of invasive BCIs; most BCI professionals hold the view that BCI users are responsible for their actions, while being uncertain regarding issues of liability; the effect of BCI activity on personal identity and self-image on the users are deemed to be unclear |
| Nijboer et al., 2014 | evaluation of BCIs among rehabilitation professionals | quant: survey qual.: focus group | | 28 rehabilitation professionals (focus group: n=28, survey: n=18) | no |  | the professionals ascribed no added value to BCI technology; human problems and practical issues should be taken into consideration; as potential BCI users those are singled out who possess intact cognition, have no extant physical movements and no sudden movements (seizures, spasms) which can cause problems |
| Pedrocchi et al., 2013 | evaluation of the MUNDUS system by testing different ATs (among them a BCI) | quant.: questionnaires (potential users) qual.: focus group (experts/ stakeholders) | | 36 potential users + 14 stakeholders (medical experts, 1 participant with ALS, 1 caregiver, 2 social enterprise representatives) + 5 participants with multiple sclerosis or spinal cord injury | yes (5 participants, mostly 2 sessions) | EEG based ERP-BCI | requirements identified by focus group: "modularity, reproduction of movements as close as possible to “natural” ones in terms of performance, preference for low encumbering device, multitask device to be used in different tasks/environments, reasonable costs and ease of use"; requirements identified by potential users: increase autonomy, enable daily activities, ease of use, comfortability; BCI were tested successfully, but are more time consuming and cumbersome than other ATs |
| Peters et al., 2016 | assessing opinions from BCI users | quant.: questionnaire | | 12 participants with severe speech and physical impairments | yes (1 session) | RSVP Keyboard™ BCI system | the participants gave highly varying feedback regarding workload, comfort, ease of use, and overall satisfaction |
| Poletti et al., 2016 | evaluation of cognitive tests via P300 BCIs | quant.: questionnaires (BDI, STAI-Y, usability questionnaire) | | 15 participants with ALS + 15 non-impaired participants (control group) | yes (1 session) | P300 BCI | participants gave positive feedback regarding usefulness and subjective experiences; feedback from control group less positive (possibly due to the differing perceptions regarding its value for the user); non-impaired participants achieved a higher processing speed level than participants with ALS |
| Riccio et al., 2011 | evaluation of two BCI applications | quant: questionnaires (NASA-TLX, VAS) | | 8 non-impaired participants | yes (1 session with 3 different tasks) | P300 BCI; BCI2000 software | no significant difference in usability could be detected between the two conditions in terms of effectiveness (level of performance), efficiency (subjective workload), and satisfaction of the use |
| Riccio et al., 2015 | testing hybrid P300-based BCI | quant: questionnaires (NASA-TLX, VAS) | | 8 non-impaired participants + 3 participants with severe motor impairments | yes (1 session) | non-hybrid P300 based BCI + hybrid P300 electro-myographic-based BCI | the hybrid system proved to be less efficient as the non-hybrid, but more accurate; it also demands a lower workload (e.g. frustration and perceived performance) but more physical effort; the participants showed a preference for the non-hybrid due to its higher usability |
| Rohm et al., 2013 | testing hybrid BCI | quant.: questionnaires (NASA-TLX, VAS) | | 1 participant with spinal cord injury | yes (43 sessions) | MI-BCI | the user reported a high workload mainly due to physical and mental strain; subjectively rated performance is better than it was in objective terms (possible reason: shared control principle leads to impression of not producing wrong commands); motivation to participate: improving quality of life by increasing independence from support staff |
| Sahinol, 2016 | BCI-research by observing two BCI laboratories for the period of three years | qual.: ethnographic field work (passive and participant observations, video and audio materials, in-depth interviews with BCI professionals and BCI users) | | 29 participants (mixed sample: participants with stroke + participants with ALS + relatives + neuro scientists + psychologists + doctors etc...) | yes (varying) | BCI2000 | thorough sociological analysis about neuroscientific practices and adaptations between human beings and their brain; postulation of a so called 'techno-cerebral subject' |
| Salisbury et al., 2016 | testing BCI among participants with spinal cord injuries | quant.: demographic questionnaire + cognitive screening qual.: semistructured qualitative questions | | 25 participants with traumatic or nontraumatic spinal cord injury | yes (number of sessions =1(?)) | Emotiv EEG system | the BCI application proved to be feasible; most participants reported joy while testing the BCI; neither cognitive test scores nor mood ratings correlated with BCI performance; however high pain ratings correlated with better BCI performance |
| Schicktanz et al., 2015 | assessing users' perspective regarding BCIs | qual.: open interviews + unsystematic discursive analysis of literature concerning experts' opinions | | 10 participants with physical impairments (ALS, muscle atrophy, para- and tetraplegia) | no |  | participants in favour of BCIs (six of ten participants) were hoping for increased autonomy and independence; participants who don’t require permanent personal assistance tended to be more skeptical and reported to fear “insufficient aims, stigmatization, and data insecurity"; compared to the experts’ position, there is only a partial overlap concerning the assessments of BCI-technology; while both groups regard independence and self-control as crucial aspects that BCIs are supposed to realize, experts think of these in terms of executing basic activities like eating or walking; the potential users, however, primarily think of independence in terms of privacy and intimacy, i.e. for example doing body hygiene and being able to be on one’s own |
| Schreuder et al., 2013 | testing two BCIs according to an user-centred design | quant.: questionnaires (QCMBCI, NASA-TLX) | | 1 participant with ischemic brain stem stroke | yes (10 sessions) | auditory and visual ERP-BCI | the user witnessed confidence in mastering and absence of fear of incompetence in BCI use; both BCI paradigms were reported as highly challenging; the reported workload was higher in using the auditory BCI (which wasn't performed successfully in contrast to the visual BCI in objective terms); quintessence: BCI paradigm should be in line with user's clinical profile |
| Taherian et al., 2016 | evaluation of Emotive EPOC | case study (no methods reported, but verbal feedback given in some form) | | 1 participant with cerebral palsy | yes (10 sessions) | commercial Emotiv EPOC BCI | control over BCI was acquired; psychological states affect BCI performance (for instance concentration problems due to personal issues bothering the participant) |
| van de Laar et al., 2010 | evaluating user experience of actual and imagined movement in BCI gaming | quant.: questionnaire | | 20 non-impaired participants | yes (2 x 2 sessions) | BCI Game BrainBasher | imagined movements prove to be more challenging since they require more concentration; actual movements produce more reliable signals and alertness is higher |
| van de Laar et al., 2013 | testing control aspect for BCI-games | quant.: questionnaire | | 158 gamers (potential BCI-gamers) | no |  | perceived control in a game depends on determinable conditions (i.e. the amount of control given); no illusion of control and no underestimation of control could be proven |
| Vansteensel et al., 2016 | testing an invasive BCI with an ALS patient | quant.: questionnaires (QUEST 2.0, PIADS) | | 1 participant with ALS (Locked-In State) | yes (67 sessions) | invasive BCI (subdural electrodes: Resume II, Medtronic) ) | the user reports being satisfied with the BCI which proves to be a viable alternative for home use |
| Vasilyev et al., 2017 | examining psychological and neurophysiological correlates of motor imagery via BCI | quant.: questionnaires ("current mood", KVIQ-20, VMIQ-2) + written interviews | | 19 non-impaired participants | yes (5 sessions) | BCI 2000 (EEG) | high variance among individuals in terms of psychological and physiological happenings related to motor imagery; high correlation between good BCI performances and vivid and detailed descriptions of the process |
| Vourvopoulos/  i Badia., 2016 | evaluation of different motor imagery paradigms in BCIs | quant.: questionnaires (PQ, VMIQ-2, NASA-TLX) | | 9 non-impaired participants | yes (3 sessions) | motor imagery based BCI (EEG) | the multimodal motor imagery paradigms request an increased workload but find acceptance among the participants; BCI control depends on the ability to deliberate control of their neural activity |
| Vuckovic/  Osuagwu, 2013 | testing motor imageries for BCI use | quant.: questionnaire (KVIQ) | | 30 non-impaired participants | no |  | the KVIQ questionnaire can select promising MI based BCI users; KVIQ results also help in finding the optimal MI strategy for BCI users |
| Won et al., 2016 | testing the impact of stimuli frequencies on SSVP-BCIs | quant.: questionnaire | | 26 non-impaired participants | yes (4 sessions) | SSCP-based BCI speller | using low frequency stimuli are reported to increase user fatigue in contrast to higher frequency stimuli |
| Zickler et al., 2009 | assessing user needs for BCIs | quant.: questionnaire (via face-to-face and phone interviews) | | 77 participants with motor impairments | no |  | participants reported to seek mobility, daily life activities, and employment; future BCIs need to realize these requests |
| Zickler et al., 2011 | testing BCI as input channel for AT software | quant: questionnaires (NASA-TLX, QUEST 2.0, VAS) qual.: open interviews | | 4 participants with physical impairments + 3 assistive technology experts | participants: yes (4 sessions) experts: no | P300 BCI | the application tested successfully, but were deemed to be unpractical for daily life use |
| Zickler et al., 2013 | evaluation of Brain Painting BCI | quant.: questionnaires (NASA TLX, QUEST 2.0, VAS, ATD PA) qual.: semistructured interviews | | 4 participants with severe motor impairments | yes (copy painting and copy spelling task + 5 painting sessions) | P300 Brain Painting | the participants attested high effectiveness, efficiency to and high sufficiency to the BCIs; they reported that they enjoyed the BCI use and gave input for further improvements of the applications |
